# Extracorporeal Shock Wave Lithotripsy and Combined Therapy in Children: Efficacy and Long-Term Results

**DOI:** 10.3389/fped.2021.609664

**Published:** 2021-05-13

**Authors:** Laura Burgos Lucena, Beatriz Fernández Bautista, Alberto Parente Hernández, Ruben Ortiz Rodríguez, Jose María Angulo Madero

**Affiliations:** Pediatric Urology, Hospital Infantil Universitario Gregorio Marañón, Madrid, Spain

**Keywords:** pediatric, urinary lithiasis, extracorporeal shock wave lithotripsy, residual fragments, combined therapy

## Abstract

**Background:** Extracorporeal shock wave lithotripsy (ESWL) is nowadays the first choice for the treatment of upper urinary tract stones smaller than 2 cm, considering its low complications and high success rate.

**Aim:** To present an update of the current situation of ESWL treatment and to analyse our series of patients and the efficacy of combined lithiasis treatment in different locations and sizes.

**Patients and Method:** Retrospective study including patients with urolithiasis treated with ESWL between 2007 and 2019. Collected data included: gender and age at treatment, presentation symptoms, imaging studies, stone location and size, complications and stone clearance. Success was defined as stone-free status or the presence of clinically insignificant residual fragments (<4 mm after 3 months follow-up). Patients with residual stones larger than 4 mm after 3 months were programmed for another ESWL session or received a combined sandwich therapy, followed by URS or percutaneous approach.

**Results:** Between 2007 and 2019, 37 patients presented a total of 41 lithiasis episodes that were treated with ESWL sessions. Median age at first procedure was 9 years old (1–17) and median follow-up time was 6 years (3–12). Stones were located in the renal pelvis, followed by the lower, middle and upper calyx, proximal ureter, and 51% of our patients had multiple lithiasis. Median stone size was 12 mm (5–45), the main component being calcium oxalate (34%). During immediate postoperative period, 8 patients (19%) presented complications: renal colic, hematuria and urinary tract infection. After the first ESWL, 41% of the patients (*n* = 17) were stone-free. Out of the 24 residual lithiasis episodes (58%), three patients (7%) underwent a second ESWL session. In the remaining 19 patients, ESWL was combined with URS or percutaneous approach to achieve complete stone clearance. Overall stone free status after combined therapy was 95% (*n* = 39).

**Conclusion:** These data support that ESWL is an effective minimally invasive technique, with low cost and morbidity, reproducible and safe for the treatment of stone disease in children. Even though lithiasis size seems to be a significant factor in ESWL success, in combination with other lithotripsy procedures it can reach very high rates of stone clearance.

## Background

The incidence of pediatric urolithiasis shows a broad discrepancy among different geographic areas. It has historically been a very frequent pathology in the Middle East Region, especially in Turkey, Pakistan, and India. Over the last decades, it has progressively increased its incidence in European countries as well as in South Asia and South America (1, 2).

There are several factors that could explain this geographic variation, such as dietary habits and bacterial prevalence. Urolithiasis is very frequently secondary to urinary tract infection (UTI), with some series reporting an incidence of 75% (3, 4). Other related factors are metabolic diseases (more frequently hypercalciuria, hyperoxaluria, and hypocitraturia) and anatomic anomalies, such as vesicoureteral reflux, bladder exstrophy, and pyeloureteral stenosis, among others.

Clinical presentation in toddlers and small infants is usually hematuria and abdominal pain, whereas in children and teenagers it more frequently entails colic lumbar pain and symptoms that are secondary to urinary tract obstruction.

Treatment approaches depend on several factors such as lithiasis size and location, stone composition, urinary tract anatomy, and available sources.

It is widely accepted that in pediatric patients, urolithiasis smaller than 4 mm can be managed with medical treatment due to the relative ease to eliminate them (5). For greater stones, numerous therapeutic modalities are available, including extracorporeal shock wave lithotripsy (ESWL) as well as endourologic approaches such as cystoscopy, percutaneous lithotomy, and ureterorenoscopy (URS). These treatment modalities are currently possible even in toddlers thanks to the miniaturization of the percutaneous approach and the improvement in URS devices.

ESWL is nowadays the first choice for the treatment of upper urinary tract stones smaller than 2 cm considering its low complications and high success rate. It was first used for the treatment of urological lithiasis in 1980 and in 1986 Newman used it in the pediatric population (6). The shock waves generated by an external lithotripter are focused on the renal stone in an attempt to fracture it and make possible its passage through the ureter and out through the urethra. During the last 30 years, lithotripters have become available in many centers worldwide, and ESWL is currently considered one of the first choices of treatment for upper urinary tract pediatric stones (7, 8). Furthermore, considering recurrences are more frequent in pediatric patients compared to adults, the use of minimally invasive treatments in this population is crucial.

During the last decades, many groups have tried to analyze successful predictive factors that allow us to establish imaging protocols and surgical indications in the different scenarios lithiasis may be found.

## Aim

The aim of our study is to present an update on the current situation of ESWL treatment and to analyze our series of patients and the efficacy of combined lithiasis treatment in different locations and sizes.

## Patients and Method

A retrospective study was performed, and it includes all our patients with urolithiasis who were treated with ESWL between 2007 and 2019. Exclusion criteria for ESWL treatment were as follows: solitary kidney, bleeding disorders, and distal ureteral or bladder lithiasis, which are rather treated by URS or cystoscopy in our hospital.

Collected data included as follows: gender and age at treatment, presentation of symptoms, anatomic anomalies, imaging studies, stone location and size, intraoperative complications, number of ESWL sessions needed, and stone clearance. Information about other treatments received for the same stone episode was reviewed.

Preoperatively, all patients were evaluated in terms of their medical history and with a physical exam, urinary analysis, coagulation, and renal function study. Imaging studies included a urinary ultrasound (US) and X-ray. Compute tomography (CT) was performed in those patients with urinary tract anomalies or when the diagnosis was unclear.

Every procedure was performed under general anesthesia, with the patient in a supine position, and antibiotic prophylaxis. Fluoroscopic control and ultrasound for radiolucent lithiasis were used for stone localization.

ESWL (Philips-Dornier Doly-S Litotripter EMSE 220) used initial power of 15 kV increasing up to 35 kV, with a pulse rate of 60 shocks per minute and a mean shock number in each session of 2,500 (700–3,200).

Postoperative follow-up includes clinical evaluation 2 weeks later and X-ray and ultrasound studies 1 and 3 months after the procedure unless any complication occurred.

Success was defined as stone-free status or the presence of clinically insignificant residual fragments (<4 mm after 3 months follow-up). Patients with residual stones larger than 4 mm after 3 months were programmed for another ESWL session. In those with initial calculi >20 mm or when residual fragments were bigger than 10 mm, ESWL has been used as combined sandwich therapy, before URS or percutaneous approach, depending on the location. For URS and percutaneous access, stone fragmentation was performed with a 2,100 nm Ho:YAG laser (Olympus Empower H35). The laser was set at 25 W (range 20-35) with a frequency of 15 Hz and 4.0 J/pulse.

For statistical study IBM-SPSS Statistics 27 was used. The chi-square test was used for categorical values and *p* < 0.05 was considered statistically significant.

## Results

Between 2007 and 2019, 37 patients presented a total of 41 lithiasis episodes that were treated with ESWL; 13 (32%) were girls and 28 boys (68%). The median age at the first procedure was 9 years old (1–17), and 14 patients (34%) were younger than 5 years old. The median follow-up time was 6 years (3–12). During the study period, one patient had two lithiasis episodes and another patient had four episodes with a period time in between them of more than 2 years (Table 1).

**Table 1:** Clinical characteristics.

**Clinical characteristics**	***n***	%^*^
Number of patients	37	-
Number of episodes	41	-
Gender		
Male	28	68
Female	13	32
Age (years)		
≤ 5	14	34
>5	27	66
Previous surgery	21	51
Previous urological pathology	8	19
Median follow-up (years)	6 (3–12)	
Side		
Right	25	61
Left	16	39
Location		
Pelvis	21	51
Inferior calyx	8	19
Middle calyx	3	7
Superior calyx	3	7
Ureter	2	5
Multiple locations	4	10
Multiple stones	21	51
Stone size		
≥20 mm	10	24
<20 mm	31	76
Complications		
UTI	3	7
Renal colic	5	12
Hematuria	5	12
Fever	3	7
Overall stone free rate	39	95

* *Percentages calculated over the total number of episodes*.

Stones were located in the renal pelvis (*n* = 22; 54%) followed by the lower renal pole (*n* = 11; 27%), middle and upper calyx (*n* = 3; 7% each), and proximal ureter (*n* = 2; 5%). A total of 21 patients had multiple stones (51%), and, in four of them (10%), there was lithiasis in multiple locations: three patients had lithiasis in the lower and middle calyx (included in lower calyx), and one had several lithiasis in the pelvis and ureter (included in pelvis location). In those who experienced lithiasis, 25 (61%) were right-sided, the median stone size was 12 mm (5–45), and 10 patients (24%) had stones >20 mm (Table 2).

**Table 2:** Lithiasis episodes classification according to its location and number.

**Location**	**Number of episodes**	**Median size (mm)**
Lower calyx	Lower calyx *n* = 8	13.5 (7.6–35)
	Low + middle calyx *n* = 3	
Medium calyx	*n* = 3	11 (10–12)
Upper calyx	*n* = 3	12.5 (10.4–14)
Pelvis	Pelvis *n* = 21	13.5 (5–45)
	Pelvis + ureter *n* = 1	
Ureter	*n* = 2	8.7 (7–10)

In total, eight of the patients (19%) presented some previous urological pathologies: vesicoureteral reflux (*n* = 2; 5%), pyeloureteral junction obstruction (*n* = 2; 5%), primary obstructive megaureter (*n* = 1), or horseshoe kidney (*n* = 1).

The stone analysis was possible in 25 cases (61%), the main component being calcium oxalate (*n* = 14; 34%), followed by calcium phosphate (*n* = 4; 10%), ammonium phosphate (*n* = 4; 10%), uric acid (*n* = 2; 5%), and xanthine (*n* = 1; 2.4%).

The most common clinical presentation was UTI (*n* = 13; 32%) followed by renal colic (*n* = 7; 17%) and hematuria (*n* = 3; 7%).

In 58% (*n* = 24) of the episodes, the lithiasis conditioned a pelvic dilation and in 10 episodes (24%) a double J catheter was placed before the ESWL session.

Regarding the diagnosis, an ultrasound study and an abdominal X-ray were performed in all patients and 17% (*n* = 7) had a preoperative CT scan.

There were no intraoperative complications, and the mean hospital stay was 24 h in 95% of the children. During immediate postoperative period, registered complications were as follows: fever (*n* = 3; 7%), urinary tract infection (*n* = 3; 7%), transient hematuria (*n* = 5; 12%), and renal colic associated with steinstrasse (*n* = 9; 22%), which was resolved without the need for new interventions. The incidence of complications and residual fragments in each location are summarized in Table 3. Furthermore, lithiasis >20 mm had significantly more residual fragments than those under 20 mm (*p* = 0.02).

**Table 3:** Complications and residual lithiasis in each location.

	***n***	**Complications**	**Residual fragments**
Lower calyx	11 (27%)	1 (*p* = 0.1)	3 (*p* = 0.2)
Medium calyx	3 (7%)	1 (*p* = 0.9)	1 (*p* = 0.3)
Upper calyx	3 (7%)	1 (*p* = 0.9)	1 (*p* = 0.3)
Pelvis	22 (54%)	4 (*p* = 0.2)	15 (*p* = 0.03)
Proximal ureter	2 (5%)	1 (*p* = 0.5)	0 (*p* = 0.8)

After the first ESWL, 41% of the episodes (*n* = 17) resulted in a stone-free condition or residual fragments smaller than 4 mm. Out of the remaining 24 lithiasis episodes (58%), three patients (7%) underwent a second ESWL session and achieve complete stone clearance. A total of 15 patients (36%), including the four patients with previous staghorn stones, had a URS procedure and all the residual fragments were eliminated. The remaining four patients (10%) went through a percutaneous procedure to become stone-free. In total, two other patients went back to their reference hospital to finish treatment. Overall, stone-free status after combined therapy was 95% (*n* = 39).

## Discussion

In the last decades, the use of ESWL for the treatment of urinary stone disease has become widely accepted. But, there still remain several interesting aspects that could be debatable.

Considering the stone location, there is controversy about the success rate with those lithiasis located in the lower renal pole. Some studies have found that, as in the adult population, this location seems to entail a lower stone-free rate (9, 10), but other groups have proven no statistically significant differences when comparing low-pole stones with those in other urinary tract locations (5, 7, 11, 12). In our hospital, ESWL is usually the first-line treatment for intrarenal stones no matter its location. In our series, patients with lower pole stones (*n* = 11; 27%) did not present more complications, and, although in this group, stone clearance after the first ESWL session was 72% (*n* = 8), the difference with other locations is not statistically significant (Table 3). However, the number of residual fragments was significantly higher in lithiasis placed in the renal pelvis, although it could be more related to the size of those stones since 6 of the 10 stones bigger than 20 mm were placed in this location (Table 3). For those lithiasis placed in the middle-distal ureter and bladder, an endoscopic approach is our treatment choice. However, some groups have also reported good results after ESWL in these locations (13).

The incidence of anatomic anomalies in our series is slightly lower than those already published, being more frequent than the VUR and PUJO (13, 14). Stone size has been considered an important predictor for ESWL success in pediatric urolithiasis. Several studies had proven that stones smaller than 1 cm have a better outcome in terms of stone clearance (15, 16). However, multiple sessions allow the achievement of good results in stones up to 3 cm in diameter (9, 17). In our experience, only two patients with lithiasis smaller than 10 mm had residual fragments after the first ESWL session. In patients with calculi >20 mm (*n* = 10; 24%), ESWL has been used as combined sandwich therapy, before URS or percutaneous approach. In all cases, an effective stone fragmentation was achieved, but the incidence of residual stones that required additional treatments was statistically significant compared to those under 20 mm. However, there were no differences in the number of postoperative complications, and only three (30%) of them presented colic pain during the first 24 h.

The youngest patient in our series was 1 year old, and up to 14 patients (34%) were younger than 5 years. Although the difference is not statistically significant, in this age group, the stone-free rate after the first session was only 36% (*n* = 5). However, it should be noted that four of the nine young children (45%) with residual stones, /ad lithiasis >20 mm before ESWL. Two of these patients presented steinstrasse during the postoperative period and total stone clearance was achieved in all of them (Table 4). As it has been reported by other groups, response to ESWL in patients under 5 years old is very good in terms of stone fragmentation and clearance, probably due to the more elastic tissues and the low body mass (18, 19).

**Table 4:** Patients under 5 years old with lithiasis >20mm.

**Calculi > 20 mm, <5 years**	**Size (mm)**	**Location**	**Complication**
Patient 1	35	Pelvis	Fever, ITU, renal colic
Patient 2	35	Lower+middle calyx	Fever, ITU, renal colic
Patient 3	33	Pelvis	Fever, ITU, hematuria, renal colic
Patient 4	24	Lower+middle calyx	No

There has been some concern about the risk of renal damage after ESWL in children. Although there may be a transient decrease in the glomerular filtration rate after the session, several groups have proven that it spontaneously recovers after 1 to 3 months. Long-term studies, including scintigraphy with DMSA and blood pressure control, have proven ESWL is safe in children and it does not associate irreversible renal damage (20, 21).

As it was previously mentioned, success was defined as stone-free status or the presence of clinically insignificant residual fragments (<4 mm after 3 months follow-up). Although some groups have reported an increased risk of recurrence in the case of residual microlithiasis (19, 22), in our series, these patients did not show earlier or more frequent lithiasis episodes compared to stone-free patients after a median follow-up of 6 years.

Recently, Machetti et al. published a multi-institutional review comparing ESWL and ureteroscopy (URS) outcomes in terms of stone clearance, emergency unit visits, and the number of general anesthetics required (23). They concluded that both treatment modalities achieve similar stone clearance but patients who underwent URS require more general anesthesia and have more complications than the ESWL patients. In our series, the stone resolution rate is similar to that published by others groups. After the first session, the stone-free rate was 41% that went up to 95% after another ESWL session or combined treatment. In our opinion, each unit protocol should be established considering the available sources and previous experience.

Eight patients (19%) presented minor complications during the first 24 postoperative hours. A total of five patients (12%) had hematuria that decreased spontaneously, three patients (*n* = 7%) had a urinary tract infection and five patients presented (*n* = 12 %) colic abdominal pain associated with steinstrasse. All of them were solved with medical treatment (hydration, analgesics, and steroid antiinflamatoires) in the next 24 h. Other groups have reported a similar rate of postoperative complications (17, 24).

In conclusion, although prospective studies with larger series would be necessary to get conclusions, in our experience, only stone size seems to be a significant factor in lithiasis clearance. In these patients combined protocols that include URS and percutaneous approach are effective and achieve a very high success rate. These data support the idea that ESWL is a minimally invasive technique, with low cost and morbidity, that is reproducible and safe for the treatment of stone disease in children.

## Data Availability Statement

The original contributions presented in the study are included in the article/supplementary material, further inquiries can be directed to the corresponding author/s.

## Ethics Statement

This study was carried out in accordance with the recommendations of Normas de manejo de pacientes pediátricos, Comité Deontológico Hospital Gregorio Marañón, and with the written informed consent of all subjects. All subjects gave written informed consent in accordance with the Declaration of Helsinki. The protocol was approved by the Comité Deontológico Hospital Gregorio Marañón.

## Author Contributions

LB was responsible for the research and article writing. BF and RO reviewed files and collected and analyzed data. AP contributed to the description and the analysis of data. JA supervised the complete work and contributed throughout all stages. All authors contributed to the article and approved the submitted version.

## Conflict of Interest

The authors declare that the research was conducted in the absence of any commercial or financial relationships that could be construed as a potential conflict of interest.
